# Association of plasma microRNA-16-5p and abdominal aortic calcification in maintenance hemodialysis patients

**DOI:** 10.1080/0886022X.2024.2368091

**Published:** 2024-07-25

**Authors:** Qiaojing Liang, Chen Fu, Yingjie Liu, Wenhu Liu, Weikang Guo

**Affiliations:** a Department of Nephrology, Faculty of Kidney Diseases, Beijing Friendship Hospital, Capital Medical University, Beijing, China; b Department of Nephrology, Beijing Jishuitan Hospital, Capital Medical University, Beijing, China

**Keywords:** Vascular calcification, hemodialysis, chronic kidney disease, microRNA, renal failure

## Abstract

Recent studies have shown that *microRNA-16-5p* (*miR-16-5p*) plays a crucial role in the pathological mechanism of vascular calcification. Nevertheless, the expression profile of *miR-16-5p* in maintenance hemodialysis (MHD) patients who are predisposed to vascular calcification remains unknown. This study aims to investigate the potential associations between calcification risk and serum *miR-16-5p* expression among MHD patients. This cross-sectional study involved 132 MHD patients from the Dialysis Center of Beijing Friendship Hospital between 1 January 2019 and 31 December 2020. The degree of calcification in MHD patients was assessed using the Abdominal aortic calcification (AAC) score, and *miR-16-5p* expression was quantified using quantitative real-time polymerase chain reaction (qRT-PCR) with the 2^–ΔΔCT^ method. Statistical analyses, including spearman correlation, linear regression and logistic regression analysis were used to explore the associations between laboratory parameters and AAC score. Calcifications were observed in 79(59.80%) patients. The linear regression showed a one-quartile decrease in miR-16-5p expression led to a significant increase in the AAC score by 5.336 (95% CI: 2.670–10.662, *p* = 0.000). Multivariate logistic regression analyses revealed that decreased miR-16-5p expression, reduced serum urea nitrogen, elevated white blood cell count, and longer dialysis vintage were significantly associated with an increased incidence of vascular calcification. The Area Under the Curve (AUC) of the Receiver Operating Characteristic (ROC) of the miR-16-5p-based logistic regression model was 0.842 (95% CI: 0.771–0.913, *p* = 0.000). There was an independent association between *miR-16-5p* expression and calcification degree. Lower *miR-16-5p* expression levels seem to be a potential risk factor of vascular calcification in MHD patients.

## Introduction

Chronic kidney disease (CKD) is a widespread disease, affecting over 10% of the general population, amounting to over 800 million individuals [1]. Maintenance hemodialysis (MHD) is a crucial treatment modality for patients with end-stage renal disease. However, patients undergoing MHD exhibit a significantly elevated mortality rate, primarily due to cardiovascular disease (CVD) complications [2]. Notably, aortic or valvular calcification, rather than atherosclerosis, poses as an independent risk factor for CVD among MHD patients [3], often leading to poor prognoses and increased complications. CKD patients frequently suffer from mineral and bone disorders, persistent inflammation states, Vitamin K deficiency, and oxidative stress, all of which contribute to the initiation and progression of vascular calcification. Consequently, the incidence of vascular calcification in CKD patients, particularly those undergoing MHD, is considerably higher than that in general population [3–5].

MicroRNAs(miRNA) are small non-coding RNAs, ranging from 18 to 25 nucleotides, that regulate gene expression and influence cellular functions [6]. Recent research has shown that miRNA play an integral role in the pathogenesis of vascular calcification [7–9]. Among them, *MicroRNA-16-5p* (*miR-16-5p*) has garnered attention due to its regulatory effects on platelet function, playing a pivotal role in acute coronary syndrome and ischemic stroke [10–12]. Notably, recent studies have reported decreased *miR-16-5p* expression in both CKD patients and animal models of the disease [8]. Transfection of vascular smooth muscle cells (VSMCs) with *miR-16-5p*-mimic has been shown to mitigate calcification through vascular endothelial growth factor A (VEGFA) signaling pathway [8]. Our prior research revealed that bone marrow mesenchymal stem cell-derived exosomes exert a crucial function in suppressing calcification by mediating the transfer of miR-15a/15b/16 and inhibiting their mutual target gene, nuclear factor of activated T cells 3 (NFATc3). This inhibition leads to downregulated osteocalcin (OCN) expression, ultimately hindering osteogenic trans-differentiation in VSMCs [13]. However, the expression of miR-16-5p in hemodialysis patients remained unknown. Therefore, the aim of this study was to investigate *miR-16-5p* expression and its relationship with vascular calcification in MHD patients.

## Materials and methods

### Study population

This cross-sectional study involved 132 MHD patients from the Dialysis Center of Beijing Friendship Hospital, Capital Medical University, from 1 January 2019, to 31 December 2020. Before the commencement of the study, all patients had undergone hemodialysis 3 times weekly for at least 6 months, with ages ranging from 18 to 80 years. Dialyzers employing high-flux polysulfone, polyethersulfone, or polymethylmethacrylate membranes with a membrane area of 1.5–1.8 m^2^ were utilized, maintaining a blood flow rate of 220–300 mL/min. Ultrapure dialysis fluid (with bacteria count <0.1 CFU/mL and endotoxin level <0.03 IU/mL) was employed, with a consistent composition: bicarbonate (35 mmol/L), acetate (3.7 mmol/L), sodium (138 mmol/L), calcium (1.75 mmol/L), magnesium (0.5 mmol/L), potassium (3.0 mmol/L), and chloride (110 mmol/L). Dialysate flow was maintained at 500 mL/min, while the dialysate temperature was consistently maintained at 36.5 °C. Anticoagulation was achieved using heparin or low-molecular-weight heparin. Exclusion criteria included: (1) Kt/*V* < 1.2; (2) severe heart failure (New York Heart Association Class III or IV), primary cardiomyopathy, malignancy, autoimmune disease, evidence of acute or chronic infection, and a history of renal transplantation or peritoneal dialysis. All participants provided informed written consent, and the study protocol was approved by the institute’s ethics committee.

The general information of subjects was recorded, including age, gender, dialysis vintage, complications. Fasting blood samples were taken before hemodialysis during the mid-week session. Routine blood tests were performed immediately including White blood cell (WBC) count, hemoglobin, serum creatinine, serum urea nitrogen (BUN), serum uric acid, serum albumin, serum calcium (corrected), serum phosphate, and intact parathyroid hormone values, serum iron, ferritin, total iron binding capacity. Corrected calcium = calcium + 0.8 × (4 – serum albumin). Then, blood samples were taken after hemodialysis to calculate Urea Reduction Ratio in dialysis (URR), URR = (BUN before dialysis-BUN after dialysis)/BUN before dialysis. Blood samples for *miR-16-5p* measurement were centrifuged, aliquoted in vials, and stored at −80 °C until assay.

### Expression levels of *miR-16-5p* measurement

#### Plasma RNA extraction

Total RNA was extracted from the prepared plasma samples (200 μl) using TRIzol reagent (Invitrogen) according to the manufacturer’s protocol. In brief, plasma sample was mixed with TRIzol reagent and chloroform. After centrifugation of the sample at 12,000 × g at 2–8 °C for 15 min, the aqueous phase was transferred to a new centrifuge tube, and equal volumes of absolute isopropanol were added. After the sample was mixed and incubated at room temperature for 15 min, it was centrifuged again at 12,000 × g at 2–8 °C for 10 min. After removal of the supernatant, the precipitate was washed in 1 mL 75% ethanol. Then, the sample was centrifuged at 7500 × g at 2–8 °C for 5 min twice. The RNA precipitate was dissolved in 14uL RNase-free water.

#### Quantitative real-time polymerase chain reaction

RNA was then reverse-transcribed using All-in-One^™^ miRNA First-Strand cDNA Synthesis Kit 2.0(GeneCopoeia(Cat.No.QP113))according to manufacturer’s protocol. The expression levels of *miR-16-5p* were quantified with the Taq Pro Universal SYBR qPCR Master Mix(Vazyme(Cat.No.Q712))according to manufacturer’s protocols by Roche LightCycler480 fluorescence quantitative PCR instrument. qRT-PCR reactions were carried out in total volume of 20ul containing 10ul 2 × Super Mix, 0.8 μL Primer(10 μM), 1 μL cDNA. The polymerase chain reaction cycling comprised predenaturation for 3 min at 95 °C, followed by 45 cycles of 10 s at 95 °C, 30 s at 60 °C, 30 s at 72 °C. Finally, with cel-miR-39-3p standard RNA as the external reference, the relative expression levels of miRNA were calculated by the 2^–ΔΔCT^ method [14].

#### Lateral lumbar radiography and evaluation of abdominal aortic calcification

All participants underwent lateral lumbar radiography. Abdominal aortic calcification (AAC) score [15] was used to evaluate calcification in MHD patients. Specifically, calcified foci along the anterior and posterior walls of the abdominal aorta adjacent to each lumbar vertebra from L1 to L4 were assessed using the midpoint of the intervertebral space. Calcifications were graded as follows: 0, no aortic calcific deposits; 1. mild calcification: <1/3 of the corresponding length of the vertebral levels; 2. medium calcification: 1/3 ∼ 2/3 of the corresponding vertebral length; 3. severe calcification: ≥2/3 of the corresponding vertebral lengths. The scores, obtained separately for the anterior and posterior walls, result in a range from 0 to 6 for each vertebral levels and 0 to 24 for the total score. All subjects were assessed independently by two investigators with blind method. The AAC score was used to determine the presence and severity of calcification, with a score of 0 indicating no calcification, 1–4 indicating mild calcification, 5–15 indicating moderate calcification, and a score of 16 or higher indicating severe calcification [16–18].

### Statistical analysis

*MiR-16-5p* expression levels were calculated as 2^–ΔΔCT^ [14]. Results are expressed as mean ± SD or median and interquartile range, depending on data distribution, for continuous variables and as frequency for categorical variables. For comparison among the groups, ANOVA analysis was used for normal-distributed continuous variables and the nonparametric test for nonnormal-distributed ones, while the *χ*^2^ test was employed for categorical variables. Kruskal Wallis analysis was used for multiple groups of continuous variables. Spearman analysis was employed to identify the factors that exhibit a correlation with AAC score and miR-16-5 expression levels. Subsequently, multiple linear regression equations were constructed to identify risk factors associated with elevated AAC score and to evaluate potential risk factors for calcification progression. Both univariable and multivariable logistic regression analysis were conducted to assess the risk factors of vascular calcification. The Area Under the Curve (AUC) of the Receiver Operating Characteristic (ROC) was utilized to assess the performance of the model. *p* less than 0.05 was considered statistically significant. Statistics were carried out using the software SPSS 23 for Windows.

## Results

### Participant characteristics

The Characteristics of the participants enrolled in this study were summarized in Table 1. The average age of the par­ticipants was 62.88 ± 13.29 years, with 76 (57.6%) being male. The average dialysis vintage was 12.33 ± 6.38 years. 79 (59.8%) patients exhibited vascular calcifications (AAC score ≥1). Compared to the non-calcification group, patients in the calcification group were significantly older (non-calcification group vs calcification group, 57.49 ± 14.11 years vs. 66.54 ± 11.42 years, *p* < 0.001). Furthermore, they had a longer duration of dialysis vintage (non-calcification group vs. calcification group, 10.77 ± 6.59 years vs. 13.37 ± 6.06 years, *p* = 0.021), higher WBC levels (non-calcification group vs. calcification group, 5.88 ± 1.78*10^9^/L vs. 6.71 ± 2.08*10^9^/L, *p* = 0.018), and lower levels of nutritional status indicators, including serum albumin (non-calcification group vs. calcification group, 39.29 ± 2.45g/L vs. 38.22 ± 3.00g/L, *p* = 0.032), BUN (25.18 ± 4.64 mmol/L vs. 22.80 ± 4.98 mmol/L, *p* = 0.007) and serum creatinine before dialysis (947.05[772.58,1120.05] umol/L vs. 862.30[709.40,999.20] umol/L, *p* = 0.028). No significant difference was observed in URR. Notably, the expression of *miR-16-5p* was significantly decreased in the calcification group compared to the non-calcification group. (non-calcification group vs. calcification group 0.95[0.41,2.92] vs. 0.62[0.33,0.89], *p = 0.002*).

**Table 1.** Characteristics of MHD patients.

	All participants	Non-calcification group	Calcification group	*p* Value
Overall (*n*)	132	53(40.2%)	79(59.8%)	
Male (*n*, %)	76(57.6)	33(62.3)	43(54.4)	0.473
Age (years, mean ± SD)	62.88 ± 13.29	57.49 ± 14.11	66.54 ± 11.42	<0.001
Serum calcium (mmol/L, mean ± SD)	2.32 ± 0.22	2.28 ± 0.21	2.35 ± 0.22	0.062
Serum phosphorus (mmol/L, mean ± SD)	1.82 ± 0.44	1.81 ± 0.46	1.82 ± 0.43	0.931
Parathyroid hormone (pg/ml, median [Q1, Q3])	207.3[118.45,347.15]	168.3[107.65,312.05]	231.40[130.6,382.60]	0.089
Serum potassium (mmol/L, mean ± SD)	4.87 ± 0.65	4.87 ± 0.53	4.87 ± 0.72	0.989
Serum albumin (g/L, mean ± SD)	38.65 ± 2.83	39.29 ± 2.45	38.22 ± 3.00	0.032
Serum ferritin (ug/L,median[Q1,Q3])	195.20 ± 112.83	175.68 ± 96.97	208.28 ± 121.16	0.172
Total iron binding capacity (umol/L,mean ± SD)	41.26 ± 7.20	41.40 ± 4.33	41.16 ± 7.77	0.853
Hemoglobin (mg/dL, mean ± SD)	114.89 ± 12.68	115.32 ± 14.02	114.61 ± 11.78	0.753
WBC count (*10^9^/L, mean ± SD)	6.38 ± 2.00	5.88 ± 1.78	6.71 ± 2.08	0.018
Serum creatinine (umol/L, median [Q1, Q3])	877.00[750.10,1062.60]	947.05[772.58,1120.05]	862.30[709.40,999.20]	0.028
Serum urea nitrogen (mmol/L, mean ± SD)	23.77 ± 4.97	25.18 ± 4.64	22.80 ± 4.98	0.007
Uric acid (umol/L, mean ± SD)	387.31 ± 72.63	405.63 ± 77.44	374.77 ± 66.81	0.018
Urea reduction ratio in dialysi s (mean ± SD)	70.48 ± 9.15	71.22 ± 6.37	69.98 ± 10.62	0.450
Dialysis vintage (years, mean ± SD)	12.33 ± 6.38	10.77 ± 6.59	13.37 ± 6.06	0.021
miR-16-5p expression (median [Q1, Q3])	0.71[0.40,1.37]	0.95[0.41,2.92]	0.62[0.33,0.89]	0.002
AAC score	3.55 ± 5.18	0 ± 0	5.94 ± 5.54	<0.001

### Correlation analysis

In the calcification group, 44 patients (33.3%) exhibited mild calcification with AAC scores ranging from 1 to 4, 29 patients (22.0%) presented with moderate calcification with AAC scores between 5 and 15, and 6 patients (4.5%) demonstrated severe calcification with AAC scores exceeding 15. Table 2 demonstrated that as the increase of the dialysis vintage duration, the degree of calcification increased, accompanied by a significant decrease in the expression level of miR-16-5p (*p* < 0.001) and serum albumin (*p* < 0.001).

**Table 2.** Characteristics of MHD patients based on AAC score.

	1 ≤ AAC score ≤ 4	5 ≤ AAC score ≤ 15	AAC score > 15	*p* Value
*N*	44	29	6	
Male (*n*, %)	25(56.8%)	15(51.7%)	3(50.0%)	0.889
Age (years, mean ± SD)	63.28 ± 11.91	70.21 ± 10.13	72.17 ± 5.85	0.056
miR-16-5p expression (median [Q1, Q3])	0.80 [0.46,1.26]	0.50 [0.27, 0.77]	0.20 [0.05, 0.48]	<0.001
Serum calcium (mmol/L, mean ± SD)	2.33 ± 0.23	2.36 ± 0.20	2.39 ± 0.23	0.734
Serum phosphorus (mmol/L,mean ± SD)	1.82 ± 0.37	1.75 ± 0.51	2.13 ± 0.29	0.157
Parathyroid hormone (pg/ml, mean ± SD)	252.50 [169.63,443.93]	175.90 [99.2, 363.80]	231.35 [123.48, 675.28]	0.343
Serum potassium (mmol/L, mean ± SD)	4.88 ± 0.69	4.71 ± 0.75	5.49 ± 0.56	0.053
Serum albumin (g/L, mean ± SD)	39.31 ± 2.54	36.92 ± 3.27	36.50 ± 0.86	<0.001
Serum ferritin (ug/L, mean ± SD)	206.40 ± 108.94	217.55 ± 145.80	177.30 ± 78.77	0.816
Total iron binding capacity (umol/L, mean ± SD)	40.63 ± 7.15	42.18 ± 8.96	40.18 ± 6.64	0.888
WBC count (*10^9^/L, mean ± SD)	6.79 ± 2.42	6.54 ± 1.59	7.01 ± 1.60	0.690
Hemoglobin (mg/dL, mean ± SD)	114.11 ± 10.55	114.59 ± 12.93	118.33 ± 15.91	0.891
Serum creatinine (umol/L, median[Q1,Q3])	854.80 [701.60,1159.60]	893.3 [739.86,985.03]	749.95 [636.55,876.25]	0.264
Serum urea nitrogen (mmol/L, mean ± SD)	23.39 ± 3.83	22.11 ± 5.48	21.71 ± 9.24	0.476
Urea reduction ratio in dialysis (mean ± SD)	70.21 ± 7.23	68.55 ± 14.75	75.26 ± 6.74	0.299
Uric acid (umol/L, mean ± SD)	378.16 ± 60.78	370.83 ± 80.57	368.17 ± 43.96	0.778
Dialysis vintage (years, mean ± SD)	11.75 ± 5.96	15.10 ± 5.78	16.83 ± 4.96	0.029

**Figure 1.**
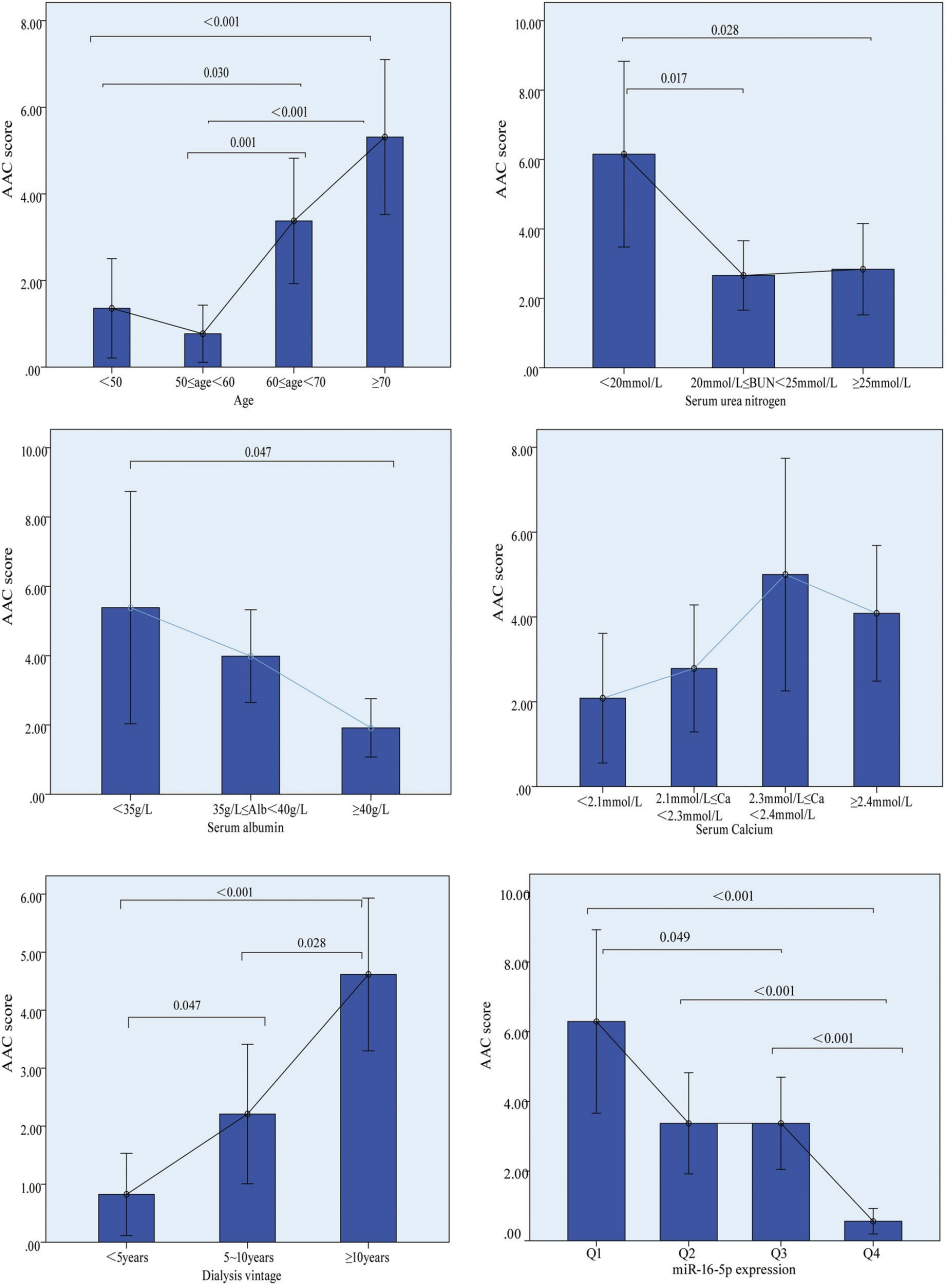
Associations between laboratory index and AAC score.

The correlation between the calcification degree-ACC score and various patient characteristics was presented in Table 3. Notably, the AAC score exhibited significant correlations with miR-16-5p expression, age, serum albumin, serum calcium, BUN, WBC counts, and dialysis vintage. Specifically, age and the duration of dialysis vintage were positively correlated with the AAC score (with correlation coefficients of 0.397 and 0.310, respectively, *p* < 0.001). Conversely, miR-16-5p expression demonstrated a negative correlation with the AAC score (correlation coefficient of −0.348, *p* < 0.001). Figure 1 further illustrated the significant associations between quartiles of miR-16-5p expression and the AAC score. However, Spearman correlation analysis demonstrated that miR-16-5p expression did not exhibit a significant association with dialysis duration, BUN levels, or WBC count. It showed a weak correlation with age and serum phosphorus levels (Table 4).

**Table 3.** Spearman’s correlation analysis of AAC score and other measurements.

Variable	Spearman’s correlation coefficient	*p*-Value
miR-16-5P expression	−0.348	<0.001
age	0.397	<0.001
Pre-dialysis BUN	−0.232	0.008
Dialysis vintage	0.310	<0.001
Serum albumin	−0.297	0.001
Serum calcium	0.182	0.037
WBC count	0.192	0.027

**Table 4.** Spearman’s correlation analysis of miR-16-5p and other measurements.

Variable	Spearman’s correlation coefficient	*p*-Value
AAC score	−0.348	<0.001
Serum phosphorous	−0.195	0.025
age	−0.213	0.015
Pre-dialysis BUN	−0.088	0.321
Dialysis vintage	−0.042	0.635
Serum albusmin	0.143	0.101
Serum calcium	0.072	0.411
WBC count	−0.050	0.567

### Regression model of calcification related factors

**Figure 2.**
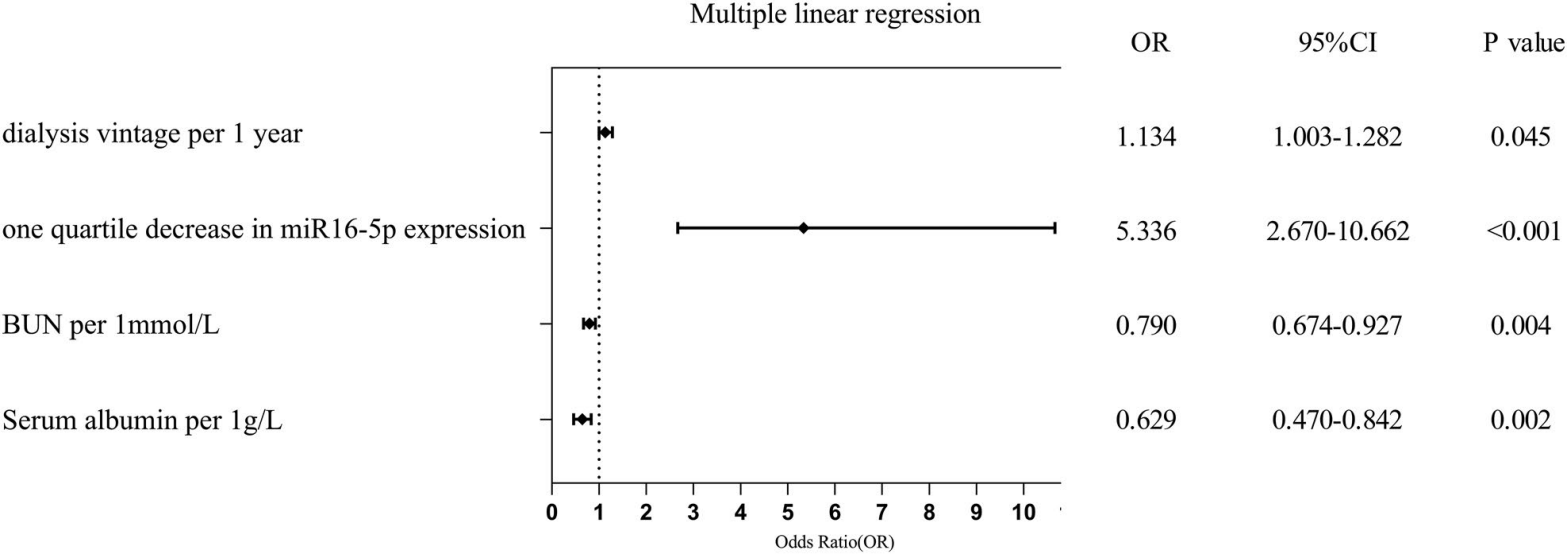
Multiple linear regression model.

We conducted a comprehensive analysis of the impact of miR-16-5p expression levels on the AAC score, employing a multivariable linear regression model. Correlation analysis identified significant associations between AAC score and various variables, including age, miR-16-5p expression, BUN, dialysis vintage, serum albumin, serum calcium, and WBC count. These variables were subsequently included into a multiple linear regression equation. It revealed that an increase in AAC score was associated with decreased miR-16-5p expression, prolonged dialysis vintage duration, reduced BUN levels, decreased albumin (*R*^2^=0.571, Durbin-Watson = 1.528, *p* < 0.001). As shown in Figure 2, a one-quartile decrease in miR-16-5p expression led to a significant increase in the AAC score by 5.336 (95% CI: 2.670–10.662, *p* < 0.001).

Logistic regression analysis was utilized to assess the risk factors of vascular calcification. The univariate regression analyses identified significant associations between vascular calcification (AAC score ≥1) and seven potential variables, including age, miR-16-5p expression, WBC count, BUN levels, pre-dialysis creatinine, dialysis vintage, and serum albumin. Subsequently, multivariate regression analyses were conducted to further investigate the risk factors associated with vascular calcification. It showed that decreased miR-16-5p expression, reduced BUN levels, elevated WBC count, and longer dialysis vintage were associated with an increased risk of vascular calcification (The Hosmer–Lemeshow test *p* = 0.206, *R*^2^=0.438, Table 5).

**Table 5.** Univariate and multivariate logistic regression analysis of vascular calcification and candidate clinical predictors.

Variable	Univariate analysis		Multivariate analysis	
	OR (95%CI)	*p*-Value	OR (95%CI)	*p*-Value
Dialysis vintage, per year	1.072 (1.009–1.139)	0.024	1.104 (1.015–1.200)	0.021
miR-16-5p expression, per unit	0.547 (0.383–0.781)	0.001	0.393 (0.224–0.691)	0.001
Age, per year	1.057 (1.026–1.089)	<0.001	1.034 (0.998–1.071)	0.064
Serum albumin, per unit	0.868 (0.761–0.990)	0.035		
WBC count, per unit	1.261 (1.036–1.534)	0.021	1.590 (1.199–2.110)	0.001
Serum creatinine, per unit	0.998 (0.997–1.000)	0.031		
Serum BUN, per unit	0.897 (0.826–0.975)	0.010	0.848 (0.753–0.955)	0.006

*Abbreviations*: CI: confidence interval; OR: odds ratio; WBC: white blood count; BUN: urea nitrogen.

**Figure 3.**
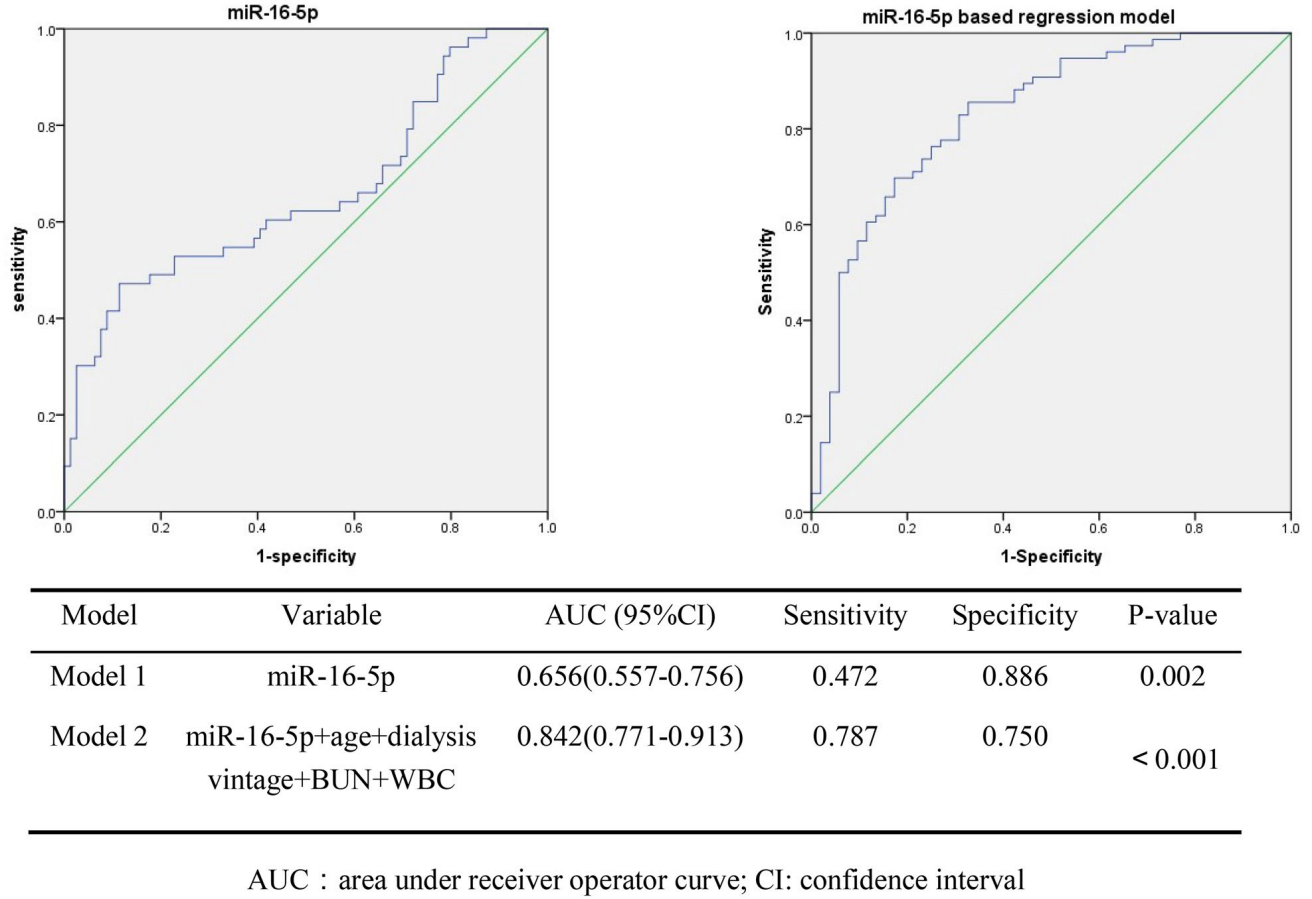
ROC curve analysis of miR-16-5p and other predictors of vascular calcification.

To evaluate the potential predictors of vascular calcification, a ROC curve analysis was performed (Figure 3). The AUC for the miR-16-5p model alone (Model 1) yielded a value of 0.656 (95% CI: 0.557–0.756, *p* = 0.002). when adjusting for confounding factors such as age, dialysis vintage, BUN, and WBC count, the AUC of the miR-16-5p-based logistic regression model (model 2) increased to 0.842 (95% CI: 0.771–0.913, *p* < 0.001).

## Discussion

In the present cross-sectional study, we demonstrated a correlation between the expression level of miR-16-5p and the severity of vascular calcification. Specifically, we found that the miR-16-5p expression level was inversely associated with the AAC score.

The *miR-16-5p* encoded by the *miR-16-1* gene, has 22 nucleotides. *MiR-16-5p* belongs to the *miR-16* family, which has an effect on anti-angiogenesis and pro-apoptosis [19]. Previous research has shown that *miR-16-5p* plays important roles in the progression of diverse malignancies, including neuroblastoma, osteosarcoma, glioma, hepatocellular carcinoma, cervical cancer, endometrial carcinoma, breast cancer, gastrointestinal cancer [20–26]. In a study of patients with acute myeloid leukemia, potential interaction network was identified involving long noncoding RNAs (lncRNA) UCA1/hsa-*miR-16-5p*/COL4A5 [27]. It had been confirmed in animal models that overexpression of *miR-16-5p* can inhibit tumor progression [28]. Additionally, *miR-16-5p* may influence platelet reactivity through noncoding regulation with PTEN, PIK3R1, CREB1, APP and MAPK1 [6]. Meanwhile, due to its regulatory effect on platelet function, *miR-16-5p* is considered a diagnostic and prognostic biomarker of acute coronary syndrome patients [10,11]. In ischemic stroke patients, *miR-16-5p* was has been predicted to be a key miRNA [12,29]. Furthermore, Takaaki Koide et al. [8] found that *miR-16-5p* expression decreased in CKD patients and CKD models.

Vascular calcification represents a significant cardiovascular complication in individuals with CKD, particularly those undergoing hemodialysis. This process is complex and influenced by various factors, as previously documented [30]. Multiple miRNAs have been implicated in this process. Specifically, Han Y [31] demonstrated that miR-223-3p inhibits interleukin-6 (IL-6)/STAT3 signaling, thereby preventing the osteogenic transition and calcification of vascular smooth muscle cells (VSMCs). Additionally, He J [9] observed a correlation between lower circulating miR-29b levels and coronary artery calcification as well as the occurrence of cardiovascular events among MHD patients. Our previous research [13,32] revealed that miR-16-5p derived from bone marrow stem cell exosomes suppresses the target gene NFATc3, leading to inhibited expression of OCN. Consequently, this inhibits osteogenic transdifferentiation and calcification of HA-VSMCs. NFATc3 belongs to the NFAT family. Furthermore, members of the NFAT family, including NFATc4, have been reported to contribute to vascular calcification by regulating the expression of osteopontin and alkaline phosphatase (AKP), thereby promoting the process [33].

Furthermore, several studies have identified that serum phosphate, calcium, parathyroid hormone play a pivotal role in regulating the calcification process. Elevated phosphorus levels have been associated with exacerbated vascular calcification in the context of CKD progression. However, recent meta-analysis conducted on adults with CKD, including both dialysis and non-dialysis patients, revealed limited clinical benefits of phosphate binders in terms of cardiovascular outcomes, including cardiovascular death, myocardial infarction, stroke or coronary artery calcification. Notably, there was no conclusive evidence that these phosphate binders effectively reduced mortality or CVD events [34]. In the present study, we observed no notable correlation between serum phosphate and the extent of calcification. This may be attributed to the fact that participants were hemodialysis patients, who were administered phosphate binders to lower serum phosphate levels. Hemodialysis itself can also effectively reduce phosphorus levels [35]. Other studies have reached the identical conclusion [36,37].

Our study revealed a correlation between lower serum albumin levels and the extent of calcification. Albumin, which is produced and secreted by liver cells, serves as an indicator of the nutritional status among hemodialysis patients. Furthermore, hypoalbuminemia indicates an inflammatory state that independently increases the risk of heart failure and acute coronary syndrome. Lower serum albumin levels also predict the severity of CVD and hospital mortality [38]. Previous studies has demonstrated that serum albumin levels play a significant role in determining the propensity for vascular calcification among patients with CKD [39,40]. Additionally, among peritoneal dialysis patients, lower albumin levels are associated with a higher risk of cardiovascular incidence [41]. In this study, the correlation analysis and linear regression revealed a significant association between the extent of calcification and BUN levels. Previous studies have revealed that pre-dialysis BUN serves as a marker for protein-energy malnutrition in MHD patients, a condition linked to heightened morbidity and mortality [42]. Elevated serum BUN levels have been correlated with enhanced survival rates in individuals undergoing chronic hemodialysis [43]. The negative correlation between BUN and the degree of vascular calcification underscores the critical role of nutritional status in the development of vascular calcification [44].

Logistic regression analysis indicated that WBC counts serve as one of the significant risk factors for vascular calcification. Recently, several researches have provided compelling evidence of a strong association between vascular calcification and inflammation [34]. Chronic microinflammation is prevalent among CKD patients, particularly those undergoing hemodialysis, and contributes significantly to cardiovascular disease and mortality. The inflammation triggers osteogenic transdifferentiation in VSMCs, thereby promoting the calcification process [45].

Recent studies have indicated that CKD, an age-related disease characterized by progressive physiological losses, impairs function and heightens susceptibility to harmful factors, including oxidative stress and inflammation. Vascular calcification, which escalates with age is a key component of this condition [30]^.^ Our study, through correlation analysis and multiple linear regression, revealed a significant association between increasing dialysis vintage and the risk of calcification. Kobayashi N. et al. [46] found that long dialysis vintage was independently associated with the increased risk of mortality in dialysis-dependent patients with critical limb ischemia undergoing revascularization. Fetuin-A, a calcification inhibitor, showed an inverse correlation with dialysis vintage among hemodialysis children [47]. Furthermore, Longer duration of dialysis vintage was associated with inflammation state, protein-energy malnutrition, and an elevated risk of CVD mortality.

In the present study, we employed the AAC score derived from lateral abdominal radiographs to assess the extent of vascular calcification. Although the 2017 KDIGO guidelines [48] recommend the use of abdominal radiographs as a reasonable alternative to computed tomography-based imaging for detecting vascular calcification in MHD patients, our method did not encompass the evaluation of coronary arteries and thoracic aorta, potentially resulting in an underestimation of the calcification severity.

Our study had several limitations. Firstly, it was conducted at a single center with a relatively small sample size. Secondly, data on patients’ medication information was not gathered, which could have provided valuable insights. Furthermore, this study was cross-sectional, meaning that the causality between risk factors and vascular calcification remains to be established through RCTs. Additionally, this study did not delve into other family members of microRNA-16 among MHD patients. In future research, we aim to further validate the expression of other microRNAs and biomarkers in MHD patients.

In summary, the present study demonstrated a negative association between miR-16-5p expression levels and the severity of vascular calcification among MHD patients. Furthermore, serum miR-16-5p levels, dialysis vintage, BUN, and WBC count were identified as independent risk factors for vascular calcification among MHD patients.
